# Barreras y facilitadores de dos proyectos piloto de telemonitoreo dirigido a pacientes con diabetes *mellitus 2*

**DOI:** 10.15446/rsap.V27n2.118508

**Published:** 2025-03-01

**Authors:** Miranda M. Ríos-Bolaños, Alide Salazar-Molina, Gabriela Nazar-Carter

**Affiliations:** 1 MR: Enf. M. Sc. Enfermería. Facultad de Enfermería, Universidad de Concepción. Concepción, Chile. mirandarios@udec.cl Universidad de Concepción Facultad de Enfermería Universidad de Concepción Concepción Chile mirandarios@udec.cl; 2 AS: Enf.-Mat. Ph. D. Enfermería. Facultad de Enfermería, Universidad de Concepción. Concepción, Chile. alisalaz@udec.cl Universidad de Concepción Facultad de Enfermería Universidad de Concepción Concepción Chile alisalaz@udec.cl; 3 GN: Psicol. Ph. D. Humanities and Social Sciences. Facultad de Ciencias Sociales, Universidad de Concepción. Concepción, Chile. gnazar@udec.cl Universidad de Concepción Facultad de Ciencias Sociales Universidad de Concepción Concepción Chile gnazar@udec.cl

**Keywords:** Telemedicina, ciencia de la implementación, diabetes *mellitus* tipo 2 *(fuente: DeCS, BIREME)*, Telemedicine, implementation science, type 2 diabetes *mellitus (source: MeSH, NLM)*

## Abstract

**Objetivo:**

Caracterizar el proceso de implementación, barreras y facilitadores de dos proyectos piloto de telemonitoreo dirigidos a pacientes con diabetes *melliuts* 2 de dos centros de salud familiar, desde la perspectiva de los profesionales de la salud participantes en el programa.

**Métodos:**

Investigación cualitativa de tipo estudio de casos. Participaron 22 directivos de salud, gestores, profesionales y técnicos involucrados en esta intervención. Se realizaron entrevistas semiestructuradas y revisión documental. La información se analizó mediante análisis de contenido y temático, guiada por el Marco Consolidado para la Investigación de la Implementación. Este estudio siguió las consideraciones éticas de E. Emanuel.

**Resultados:**

Las principales barreras fueron falta de participación en toma de decisiones, planificación deficiente, complejidad del proceso, recursos escasos, conectividad deficiente, vinculación entre dispositivos electrónicos deficiente y plataforma poco amigable. Los facilitadores se centraron en actitudes positivas hacia la intervención y la utilidad percibida para el manejo de las condiciones crónicas.

**Conclusiones:**

Las principales barreras detectadas aluden a la gestión del proceso de implementación y aspectos tecnológicos, mientras que los facilitadores más destacados fueron de tipo actitudinal. Esta información podrá ser referente para futuras investigaciones que busquen aplicar herramientas digitales en diversos centros de salud.

## Abstract (en)

**Objective:**

To characterize the implementation process, barriers, and facilitators of two pilot telemonitoring projects targeting patients with type 2 diabetes *mellitus* from two family health centers, from the perspective of healthcare professionals participating in the program.

**Methods:**

This qualitative case study included 22 participants: health directors, managers, professionals, and technicians involved in the intervention. Data was collected through semi-structured interviews and document review. The information was analyzed using content and thematic analysis guided by the Consolidated Framework for Implementation Research. Ethical considerations outlined by E. Emanuel were followed.

**Results:**

The main barriers included lack of involvement in decision-making, poor planning, process complexity, limited resources, poor connectivity, insufficient integration between electronic devices, and an unfriendly platform. Facilitators were centered on positive attitudes toward the intervention and the perceived usefulness for managing chronic conditions.

**Conclusions:**

The primary barriers identified were related to the management of the implementation process and technological aspects, while the most notable facilitators were attitudinal. This information could serve as a reference for future studies aiming to implement digital tools in various healthcare settings.

Las enfermedades no transmisibles (ENT) son la principal causa de muerte a nivel mundial, representando el 74% de los decesos, y en Chile se asocian al 85% de las defunciones, incluyendo un 3% atribuible a la diabetes *mellitus* tipo 2 (DM2) 1. Según la Encuesta Nacional de Salud 2016-2017, la prevalencia de DM2 es del 12,3%, sin embargo, solo el 42,9% está compensada, lo que agrava los efectos de esta enfermedad 2.

La Organización Mundial de la Salud (OMS) ha recomendado la implementación de la salud digital para el manejo de personas con enfermedades crónicas, destacando el telemonitoreo como una herramienta eficaz 3. Esta estrategia permite el control remoto de los parámetros biométricos, demostrando su eficacia al reducir costos y tiempos, aliviar la congestión en centros de salud y disminuir tasas de mortalidad 4; además, mejora la adherencia al tratamiento de los pacientes, satisfaciendo sus necesidades de autonomía desde sus hogares 4. Contribuye asimismo a la equidad en el acceso a la salud, facilita el trabajo multidisciplinario, garantiza la continuidad del cuidado y fortalece la relación entre el equipo de salud y los pacientes 4. Aunque existe evidencia sustantiva de los efectos positivos del telemonitoreo, su implementación en la práctica clínica continúa siendo un desafío, lo que afecta su correcta implementación por factores tales como la falta de evaluación de intervenciones, poca integración en centros de salud y la resistencia de los pacientes 5-6.

El Ministerio de Salud de Chile (MINSAL), en colaboración con el Gobierno de Corea del Sur, implementó un proyecto piloto de telemonitoreo para pacientes con DM2, en dos Centros de Salud Familiar (CESFAM) en el centro-sur del país 7-8. El proyecto utilizó medidores de glicemia y presión arterial, un reloj inteligente y una aplicación móvil, para transmitir información al CESFAM, que luego era analizada por un equipo de salud liderado por enfermeras.

El Marco Consolidado para la Investigación sobre la Implementación (CFIR por su sigla en inglés) corresponde a un modelo metateórico creado para la evaluación de la implementación de intervenciones. Se ha utilizado ampliamente en la investigación de servicios de salud para guiar evaluaciones formativas sobre barreras y facilitadores de la implementación de intervenciones de eHealth 9, a partir de una propuesta de cinco dominios y 39 constructos.

Este estudio se planteó como objetivo caracterizar el proceso de implementación, las barreras y los facilitadores de dos proyectos piloto de telemonitoreo dirigidos a pacientes con DM2 de dos centros de salud familiar, desde la perspectiva de los profesionales de la salud participantes del programa, utilizando el CFIR como marco de evaluación.

## MÉTODO

Estudio cualitativo de tipo estudio de caso de dos proyectos piloto de telemonitoreo dirigidos a pacientes con DM2 implementados en dos CESFAM de las regiones Metropolitana y del Biobío, en Chile, y desarrollados entre diciembre de 2017 y diciembre de 2018.

En el estudio participaron directivos, gestores, profesionales y técnicos involucrados en el diseño o la implementación de los programas. Se identificaron 29 informantes claves, de ellos, 22 accedieron a participar: cuatro participantes (dos médicos, una nutricionista y un tecnólogo médico) pertenecían al MINSAL (nivel central), tres participantes (dos profesionales de enfermería y un informático) pertenecían a los servicios de salud (nivel intermedio) y 15 profesionales (seis enfermeros, tres odontólogos, dos técnicos en enfermería, una asistente social, una kinesióloga, una matrona y un médico) se desempeñaban en CESFAM (nivel local).

Se emplearon entrevistas semiestructuradas y revisión documental como técnicas de recolección de información. Las entrevistas se realizaron entre junio de 2022 y febrero de 2023, en una única sesión por participante. Previamente, se diseñó un guion de entrevista basado en el CFIR, el que fue evaluado por una experta en salud digital en cuanto a claridad, precisión, pertinencia y suficiencia de las preguntas. Las entrevistas fueron realizadas por una o dos integrantes del equipo de investigación, se llevaron a cabo de manera online o presencial en las instalaciones del CESFAM, con duración aproximada de 40 minutos. Cada entrevista fue grabada y transcrita en su totalidad.

Para la revisión documental se identificó toda la documentación del proyecto a la que se pudo tener acceso, ya fuera por medio de informantes clave o búsqueda en internet. Se analizaron en su totalidad cuatro documentos de tipo informes y documentos de trabajo 7-11.

### Análisis de datos

Posteriormente a la transcripción de las entrevistas, se realizó análisis de contenido 12. En una primera instancia se identificaron los aspectos emergentes en el relato de los participantes. Este método permitió identificar, organizar y analizar patrones o temas de los relatos, dando la posibilidad de comprender el fenómeno de estudio, a partir de una minuciosa y reiterada lectura de la información. La codificación de los datos fue realizada por la investigadora responsable y revisada por las coautoras. Los temas principales fueron guiados por el CFIR, pero los datos fueron identificados a raíz de la información brindada por los participantes. Seguidamente, se realizó un análisis de contenido a través de la revisión documental.

Para cautelar el rigor metodológico del proceso de investigación cualitativa, se atendió a los criterios de credibilidad, auditabilidad y transferibilidad 13. Para resguardar el cumplimiento de estos criterios se mantuvieron las notas de campo generadas durante la recogida de la información, grabación de las entrevistas en audio, transcripción textual de las entrevistas, descripción detallada de las características de los participantes, triangulación entre investigadores y con la teoría.

Durante el desarrollo de la investigación se respetaron los principios éticos propuestos por Emanuel 14 y los aspectos contenidos en las pautas del Consejo de Organizaciones Internacionales de las Ciencias Médicas (CIOMS). El estudio recibió la aprobación de los comités de ética de los servicios de salud correspondientes (SST Acta No. 68 y SSMOC Carta No. 55) y todos los participantes firmaron un consentimiento informado.

## RESULTADOS

### Caracterización del proceso de implementación del proyecto piloto de telemonitoreo para pacientes con diabetes *mellitus* tipo 2

Se identificaron tres etapas en el proceso de implementación del proyecto piloto de telemonitoreo. El Cuadro 1 describe el proceso de implementación, sus etapas, actividades y participantes. Las letras en superíndice dentro del cuadro indican los responsables y los participantes de cada actividad en las distintas etapas del proyecto.

**Cuadro 1:** Etapas, actividades y responsables del proceso de implementación del proyecto piloto de telemonitoreo para pacientes con diabetes mellitus tipo 2

I. Etapa de diseño	II. Etapa de implementación	III. Etapa de cierre
1. Solicitud de propuesta del proyecto Knowledge Sharing Process (KSP) de telemedicina(a, b) 2. Firma del convenio (a-d) 3. Visita técnica a Corea del equipo MINSAL (c,d) 4. Definición de criterios de Selección (d) - Servicios de salud participantes - Proceso clínico y tecnología por implementar - Pacientes beneficiarios	1. Capacitación a los equipos encargados de la implementación (c, e-f) - Funcionamiento del piloto - Dispositivos clínicos y plataforma web para el monitoreo 2. Reclutamiento y capacitación de pacientes participantes (f-h) - Pacientes que cumplan con criterios de inclusión 3. Ejecución de la Intervención(f-i) - Entrega de dispositivos clínicos a pacientes participantes - Taller grupal para la capacitación de los pacientes en el uso de los dispositivos clínicos - Monitoreo a través de plataforma web por parte del equipo de salud - Llamado telefónico por enfermera o Salud Responde en caso de alertas del sistema - Atención presencial en el CESFAM para resolución de problemas - Evaluación y retroalimentación a pacientes participantes	1. Devolución de dispositivos clínicos (f-h) - Solicitud devolución de los dispositivos clínicos a los participantes 2. Elaboración y entrega de informes (e,f) -Entrega informe final de resultados clínicos de pacientes participantes 3. Clausura del convenio Chile-Corea (a-e)
Responsables: a. Gobierno de Chile. b. Gobierno de Corea. c. Equipo de investigación coreano. d. Profesionales referentes MINSAL. e. Profesionales referentes de los Servicios de Salud. f. Profesional referente Programa de Salud Cardiovascular. g. Equipo multidisciplinario del CESFAM. h. Pacientes seleccionados. i. Equipo de Salud Responde.		

### Barreras y facilitadores de la implementación del proyecto piloto de telemonitoreo basados en el CFIR

La exposición de los resultados se estructuró conforme a los dominios y constructos del CFIR. De estos últimos, 17 fueron abordados en profundidad y aparecieron de manera explícita en las entrevistas y en la revisión documental.

### Dominio 1. Características de la intervención

#### Origen de la intervención

Todos los entrevistados concluyeron que el origen de la intervención fue de carácter externo. La intervención fue descrita como "impuesta" por el nivel central sin posibilidad de negociación por el centro de salud: "No nos preguntaron, había que hacerlo. Esto hay que hacerlo, necesitamos que ustedes seleccionen a los pacientes que van a participar, estos son los tiempos, no nos preguntan si ustedes quieren participar, era algo que había que hacerlo" (Profesional de Enfermería, CESFAM).

#### Calidad y fuerza de la evidencia

Los participantes tenían nociones generales del telemonitoreo y sus efectos. Respecto a la percepción de sus resultados, estos fueron disímiles; algunos participantes indicaron que "no había buenos resultados" y otros que inicialmente había buenos resultados, pero luego "se desilusionaban": "Yo tenía más o menos indicio de otras estrategias que se habían realizado en otros servicios de salud, pero no había buenos resultados" (Profesional de Enfermería, SS).

#### Adaptabilidad

El intercambio de información entre el equipo chileno y el coreano se vio limitado por el idioma, aspecto mencionado de forma recurrente por el equipo gestor: "El idioma fue un tema complejo en la implementación porque todo lo que conversábamos era mediante intérprete, por lo tanto, puede que algunos detalles hayan quedado no muy claros" (Profesional MINSAL).

Así mismo, no existía una vinculación de la plataforma coreana con los sistemas de los centros de salud: "Era una plataforma paralela, no había conexión con la del centro de salud, eso también era un quiebre para los profesionales de salud, porque si hubiese sido una sola plataforma, habría funcionado mucho mejor, vinculado a los sistemas que tienen hoy en día los centros" (Profesional MINSAL).

#### Complejidad

La percepción de complejidad del proyecto fue compartida por varios participantes. Las negociaciones, los tiempos acotados, la falta de claridad en la información entregada, la inexperiencia del usuario en el manejo de los dispositivos clínicos, un software limitado y el esfuerzo adicional por el equipo de salud al no contar con un apoyo extra, complejizaron la implementación del telemonitoreo. De forma específica, los participantes relataron que las negociaciones con el equipo de Corea fueron complejas: "Las gestiones con el gobierno de Corea fueron complicadas en cierto punto. Al final era como cumplir con el gobierno de Corea, hagámoslo, porque era un compromiso, pero incluso para nosotros estaba significando algo más complicado de lo que se pensó" (Profesional MINSAL).

Los participantes describieron falta de apoyo adicional para la ejecución del proyecto, por lo que se requirió un gran esfuerzo del equipo de salud: "Hicimos todos los esfuerzos humanos para sacar adelante el proyecto, pero eso tiene mucha consecuencia en los desgastes de los equipos" (Profesional MINSAL).

#### Ventaja relativa

La intervención de telemonitoreo se reconoce como una oportunidad para el cambio en el enfoque en la atención, tendiente a dar protagonismo al usuario, informarse de su enfermedad, mejorar la adherencia al tratamiento y finalmente mejorar su condición de salud.

Yo lo vi como una oportunidad porque claramente era otra forma de enfoque para el manejo de su patología, porque muchas veces el paciente también reclama que siempre es lo mismo, control con nutricionista que les va a decir lo mismo, que los medicamentos, que tienen que tomar tantos fármacos, entonces era una manera nueva para que ellos también se reactivaran y se adhieran un poco más a los controles entonces les podía hacer más llamativo (Profesional de Enfermería, CESFAM).

### Dominio 2. Contexto externo

#### Necesidades y recursos de los pacientes

El telemonitoreo se percibió como una herramienta que permitía al usuario satisfacer la necesidad de respuesta ante una emergencia de salud: "Para el equipo, para las personas sí fue beneficioso, imagínense tener a alguien ahí que antes no existía que estuviera constantemente disponible para dar respuesta a tu emergencia de salud" (Médico, CESFAM).

### Dominio 3. Contexto interno

#### Características estructurales

Los participantes mencionaron que al ser una organización jerárquica, el establecimiento de salud debió cumplir con el mandato de la implementación del proyecto piloto de telemonitoreo: "Como éramos dependientes del Servicio no nos podíamos negar a hacerlo, se genera una obligación de trabajar en este proyecto, el Servicio de Salud es elegido como piloto para los proyectos del MINSAL, entonces finalmente somos como un CESFAM como conejillo de indias para probar estas cosas" (Profesional de Enfermería, CESFAM).

Algunos informantes claves percibieron que la cultura de trabajo del establecimiento de salud no favorecía el trabajo en equipo para la implementación de este tipo de estrategias: "Yo tengo que declarar que lamentablemente somos un CESFAM que trabaja bien como isla, por lo tanto, cuando llega un proyecto o llega algo, la gente le coloca nombre en el CESFAM y quizás no es tan trabajo en equipo con lo que se va a implementar" (Profesional de Enfermería, CESFAM).

#### Clima de implementación

La experiencia de tener que dejar otros temas a un lado por cumplir con la implementación generaba tensión en el equipo: "Uno lo ve, siempre con una estrategia nueva hay sobrecarga de trabajo, siempre. Si yo hago otra cosa, voy dejando a un lado otros temas después nadie te cubre mucho en eso, entonces también es una sobrecarga" (Profesional MINSAL).

En relación con los incentivos, se identifica la ausencia de reconocimiento por el trabajo realizado en la implementación del telemonitoreo: "De eso me estaba tratando de acordar, parece que no tuve ni una hoja de mérito, finalmente me dijeron gracias, eso sí". (Informático, SS). "No ningún incentivo, era parte de nuestro rol" (Profesional MINSAL).

#### Preparación para la implementación

Algunos entrevistados mencionan que los directivos y las autoridades no estuvieron realmente involucrados en el proyecto y sus actividades: "No hubo apoyo por parte de ellos, solo fue al principio y ya después desaparecieron.

Jamás nadie nos preguntó si estábamos bien, si teníamos dificultades, si requerimos alguna ayuda con algo, no" (Profesional de Enfermería, CESFAM).

#### Recursos disponibles

Todos los entrevistados concordaron en que existía escasez de recurso humano para la implementación del proyecto piloto de telemonitoreo: "El recurso humano no era el suficiente, había que dejar cosas de lado para poner horas en el proyecto, no tenía recurso humano asociado, esa era una de las grandes brechas porque se tuvo que usar recurso humano del mismo establecimiento. Eso no dio abasto y se tuvieron que readecuar agendas" (Profesional de Enfermería, CESFAM).

Con respecto al recurso tecnológico, fue evidenciado en los relatos de los informantes claves los problemas de conectividad en los sectores asignados para el telemonitoreo: "En el CESFAM hay harta gente en lugares rurales y apartados, entonces la señal telefónica en esos lugares era mala. Si bien se comunicaba por bluetooth, la información no se veía reflejada hasta que el teléfono tomara señal" (Informático, SS).

### Dominio 4. Individuos involucrados

#### Conocimientos y creencias

Los relatos reflejan diferentes percepciones acerca del proyecto piloto de telemonitoreo, desde opiniones positivas y entusiastas hasta cierto grado de escepticismo o preocupación acerca de su implementación y efectividad: "Yo siempre pensé que podía funcionar, porque la idea era pacientes descompensados, entonces uno sabe que a un paciente descompensado uno le aporta un poquito, tiende mejorarse" (Profesional de Enfermería, SS). "Era algo nuevo, no se había visto en otros lados. En realidad, era todo nuevo, no sabíamos si iba a resultar o no, tampoco estaban las directrices muy claras, se tuvo que partir de cero desde un protocolo" (Profesional de Enfermería, CESFAM).

### Dominio 5. Proceso de implementación

#### Planificación

El tema más recurrente entre los entrevistados fueron los tiempos limitados, en particular, la falta de planificación debido al corto tiempo designado para ello: "La única sensación que tengo es que estábamos eternamente para empezar y costó mucho empezar, yo creo que fue como un año de pura planificación, revisión y todo eso. De hecho, estábamos atrasados" (Profesional de Enfermería, SS).

Así mismo, se recalcó la falta de planificación al elegir al grupo objetivo para la implementación del proyecto: "Desde mi punto de vista, hubo una mala planeación con el tema etario de las personas porque algunas personas no contaban con un smartphone y tampoco habían usado un smartphone en su vida" (Informático, SS).

#### Involucramiento o atracción

Para algunos miembros del equipo de salud hizo falta motivación para poder llevar a cabo la implementación de la intervención: "No hubo ninguna motivación. Entonces por supuesto cada uno tiene que cumplir sus metas y esto no estaba dentro de las metas del CESFAM" (Profesional de Enfermería, CESFAM).

#### Ejecución

Se evidenció la falta de fidelidad de la implementación con el curso de acción planificado, del cumplimiento del cronograma establecido y del compromiso de aquellos involucrados en el proceso de implementación. Se realizaron cambios en el camino debido a limitaciones presupuestarias o la necesidad de recursos adicionales: "Tuvimos que pedirle al Gobierno de Corea que aportará con el plan de datos por ejemplo, porque o sino no podíamos, los fondos no venían dentro del proyecto original, venía la tecnología, los dispositivos, la plataforma pero el tema del internet ... claro, el tema del internet en este país no es gratuita para todos" (Profesional MINSAL).

#### Reflexión y evaluación

La percepción general del equipo involucrado en implementar el proyecto piloto de telemonitoreo fue que no hubo una retroalimentación del progreso o una evaluación final: "Yo no tengo conocimiento de si en algún momento vinieron a evaluar o refirieron nuestra compensación aumentó en tanto porcentaje o hicimos un estudio" (Profesional de Enfermería, CESFAM).

Al finalizar el proyecto piloto, la experiencia para algunos, desde distintas perspectivas, es que resultó ser un problema más a los que habitualmente se enfrentaban en el servicio público: "Para mí al final cuando ya terminó todo, era un 'cacho', se transformó al final en un 'cacho' porque seguían pidiendo cosas, ellos querían cosas, que nosotros no les podíamos dar, desde el punto de vista con Corea, eso fue agotador, las interacciones con ellos" (Profesional MINSAL).

#### Líderes

En algunos relatos del equipo de implementación, se menciona la importancia de contar con líderes y directivos comprometidos y motivados con la innovación: "En los años que estaba el Doctor como director, él nos incentivó mucho al trabajo como equipo, y eso cuando tienes una mirada de los directivos distinta, hace más permeable este tipo de cosas" (Profesional de Enfermería, CESFAM).

Los Cuadros 2 y 3 presentan una síntesis de las barreras y los facilitadores del proceso de implementación del proyecto piloto de telemonitoreo.

**Cuadro 2:** Síntesis de las barreras del proceso de implementación del proyecto piloto de telemonitoreo

Barreras de la implementación del proyecto piloto de telemonitoreo
De gestión
1. Falta de participación en la decisión de implementar el proyecto piloto de telemonitoreo 2. Poca socialización y claridad del objetivo del proyecto piloto de telemonitoreo 3. Establecimientos con organización interna jerárquica 4. Planificación deficiente de la ejecución del proyecto 5. Poca claridad de roles en el equipo ejecutor 6. Complejidad: tiempos limitados para la implementación del proyecto. Asignación de funciones adicionales a las habituales 7. Escasez de recurso humano para la ejecución y seguimiento del proyecto 8. Falta de recursos económicos para contratación de personal asistente 9. Sobrecarga de trabajo en equipo ejecutor 10. Poco involucramiento, apoyo y compromiso por parte de las autoridades y directivos 11. Falta de trabajo en equipo en los centros de salud 12. Falta de evaluación, reflexión y retroalimentación del proyecto piloto de telemonitoreo 13. Falta de conocimiento previo de intervenciones de telemonitoreo 14. Falta de incentivos y reconocimiento a la participación 15. Falta de estrategias de motivación de parte de los directivos hacia el equipo ejecutor 16. Escasa información de políticas externas que impulsen las estrategias digitales en salud
Técnicas
1. Conectividad deficiente 2. Vinculación deficiente entre dispositivos clínicos 3. Plataforma para el monitoreo poco amigable 4. Poca adaptabilidad con sistemas informáticos chilenos 5. Poca flexibilidad para cambios en la plataforma 6. Idioma de la contraparte dificultó la comunicación 7. Dispositivos clínicos no certificados en el país 8. Falta de asesoría técnica

**Cuadro 2:** Síntesis de los facilitadores del proceso de implementación del proyecto piloto de telemonitoreo

Facilitadores de la implementación del proyecto piloto de telemonitoreo
Actitudinales 1. Actitud favorable hacia la intervención de parte del equipo gestor 2. Percepción de herramienta útil para el control de los pacientes 3. Percepción de beneficio para el usuario 4. Líderes comprometidos 5. Espacio para la innovación Técnicos 1. Dispositivos clínicos de fácil manejo

## DISCUSIÓN

Los resultados de esta investigación muestran la percepción de los participantes del diseño y la implementación una intervención de telemonitoreo dirigido a pacientes con DM2.

Los informantes clave coinciden en que una barrera fue el ser una iniciativa centralizada con escasa participación local. Damschroder 9 destaca que la probabilidad de éxito en la implementación disminuye cuando se toman decisiones sin involucrar activamente a las personas que serán encargadas de su implementación. La falta de inclusión del equipo ejecutor en la decisión de implementar el proyecto piloto y la estructura jerarquizada del Sistema de Salud chileno pueden haber afectado la apropiación del proyecto. Esto podría haber llevado a que funcionara de manera independiente de la atención habitual en los CESFAM, en lugar de integrarse a las prestaciones. Este fenómeno se alinea con un estudio en Noruega que evaluó la adopción de una intervención de eHealth en un hospital, donde se observó que, en unidades con jefaturas jerárquicas, la adopción fue baja, mientras que en aquellas con mayor autonomía se facilitó la integración 15.

La falta de planificación fue identificada como otra barrera, evidenciada en los tiempos limitados para la implementación, la poca claridad y la falta de información en los objetivos del proyecto piloto, la escasa claridad de roles y los cambios de pacientes durante la duración de la intervención. Este hallazgo es similar a lo reportado por Seljelid 16, quien afirmo que la falta de información y de asignación de responsabilidades podría reducir potencialmente la motivación de los involucrados.

La complejidad del proceso del proyecto piloto de telemonitoreo fue otro factor obstaculizador. La adición de nuevas actividades a las funciones habituales generó sobrecarga de trabajo, estrés y presión en el equipo. Ello es consistente con los reportes de estudios que han evaluado intervenciones en eHealth en pacientes crónicos, que señalan que la adaptabilidad y el grado de complejidad de una estrategia pueden generar un impacto negativo en su implementación 17-19.

La falta de recurso humano fue identificada como una barrera en la implementación, generando sobrecarga de trabajo y desgaste en algunos miembros el equipo ejecutor. Al igual que en el presente estudio, otros han identificado la necesidad de contar con recurso humano que permita que estrategias innovadoras se instalen efectivamente 17,20-21. Kirkland 22 señala que el aumento de la carga laboral para satisfacer las necesidades de los pacientes del proyecto genera agotamiento físico y mental en el equipo ejecutor.

El ejercicio del liderazgo apareció como estratégico en el proceso evaluado. Los líderes son considerados proveedores clave de nuevos conocimientos e influencia fundamental en las iniciativas de implementación, especialmente en el ámbito de eHealth 23. Su compromiso resulta vital para la adopción de soluciones digitales en salud ya que implica respaldar activamente la implementación, proporcionar recursos, fomentar una cultura favorable al cambio y establecer políticas y procedimientos adecuados 24.

En uno de los centros de salud se evidenció cómo el apoyo y la preocupación de los líderes incentivaron el trabajo en equipo, facilitando que se llevara a cabo el proyecto piloto. Esta percepción concuerda con los siete roles de los líderes definidos por Laukka 24: apoyo, gestor de cambio, defensor, gestor de proyectos, toma de decisiones, facilitador y campeón. El apoyo en la forma de suministro de recursos para la implementación de la intervención (económicos, capacitación) es clave para el éxito de un proceso de cambio 25. Así también, el líder debiera identificar cualquier resistencia e informar a todos los involucrados sobre los cambios esperados 26.

En la misma línea, Erlingsdottir 27 plantea que el verdadero factor del éxito de una implementación radica en la adopción de un liderazgo descentralizado y en el empoderamiento del personal a través de una mayor capacidad para el autoliderazgo.

Para garantizar la implementación exitosa de la innovación y lograr los resultados deseados, es fundamental llevar a cabo de manera efectiva los planes y las tareas de implementación 9. En el caso del proyecto piloto de telemonitoreo, se hicieron cambios durante su ejecución debido a limitaciones presupuestarias o la necesidad de recursos adicionales que no habían sido considerados en la planificación inicial. Todo lo anterior incidió en la percepción de continuidad del proyecto y por ende en el grado de compromiso de los participantes.

Otro factor percibido por los informantes clave fue falta de evaluación de la intervención. La ausencia de mecanismos de evaluación y retroalimentación obstaculizan la capacidad de las partes interesadas para reflexionar sobre el proceso de implementación y realizar los ajustes necesarios, tanto a nivel organizacional como tecnológico, humano, social, ético-legal y costos asociados 6.

Adicionalmente, fue referido como un obstaculizador la falta de conocimiento de estrategias implementadas en eHealth en el país 11,28, de los cuales no se conocen sus resultados ni se dispone de antecedentes nacionales que respalden la implementación de este tipo de estrategias y entreguen orientaciones para su implementación. Está reportado que la falta de conocimiento previo de estrategias similares puede generar dudas sobre la eficacia de la intervención y limitar su aceptación y adopción (29), lo cual coincide con los resultados del presente estudio.

Algunos factores tecnológicos, tales como la vinculación de los dispositivos clínicos y la conectividad deficiente para la transferencia de datos, obstaculizaron el proceso de implementación. Existe evidencia de que el soporte tecnológico afecta la viabilidad de los programas 22. Un estudio realizado en tres hospitales de Países Bajos, que buscaba evaluar la implementación del monitoreo continuo utilizando sensores portátiles inalámbricos 30, reveló cómo una deficiente conexión a Wi-Fi ocasionó la pérdida de datos y la discontinuidad del proyecto en dos hospitales.

La falta de adaptabilidad de la intervención a las necesidades locales dificultó su implementación, debido a las dificultades de comunicación con el equipo coreano resultado de las diferencias idiomáticas y a que la plataforma web del monitoreo no estaba conectada con los sistemas actuales de los CESFAM. Este tipo de barrera ha sido evidenciada en otros estudios, en los que la falta de adaptabilidad generó frustración por el tiempo y el esfuerzo extra requerido para el manejo de dos plataformas en paralelo 17,20,31-32.

A pesar de las barreras mencionadas, el telemonitoreo se percibe como ventajoso en comparación con la atención convencional. Este enfoque permitiría la participación de los pacientes en su cuidado al registrar y reportar regularmente datos de salud, fomentando la responsabilidad y la autogestión. Además, supera barreras geográficas, mejorando el acceso a la atención, especialmente en zonas rurales o distantes, y beneficiando a pacientes con discapacidades o dependencias al evitar visitas presenciales a los centros de salud. Este hallazgo coincide con estudios sobre la percepción de profesionales que emplean eHealth para el manejo de enfermedades crónicas 17,19,33.

Es importante destacar que la identificación de las necesidades y los recursos de los pacientes por parte del equipo ejecutor fue un facilitador para la adopción de la estrategia digital. El telemonitoreo ofrece una forma de seguimiento continua y en tiempo real de los pacientes con DM2, por lo cual se percibió como una herramienta útil para satisfacer las necesidades de los pacientes y mejorar su calidad de vida, generando una positiva aceptación 34-35.

Es esencial considerar las creencias, las actitudes y los conocimientos de los involucrados en una intervención, ya que influyen en la aceptación y en el compromiso 29. Estos aspectos pueden ser moldeados por la educación, la experiencia previa, las interacciones sociales y las percepciones culturales. Actitudes positivas, como entusiasmo y motivación, favorecen la participación 29,32. En este estudio se notó una actitud general positiva entre los participantes, basada en la creencia en la utilidad de intervenciones eHealth, como el telemonitoreo, para el cuidado de enfermedades crónicas.

La oportunidad de innovar fue otro facilitador presente en los relatos de los informantes claves. Las mejoras en salud ofrecen tratamientos avanzados, accesibilidad mejorada, coordinación eficiente, reducción de costos y concienciación para estilos de vida saludables. El espacio innovador permite la exploración de ideas, fomentando mejoras y transformaciones en atención médica y salud digital 36. Bates 37 destaca consideraciones previas a la implementación: recursos económicos y físicos, participación profesional y pacientes, información sobre propiedad intelectual, coordinación con expertos en software, visión a largo plazo y priorización estratégica de la innovación.

### Aportes de la investigación

Esta investigación hace un aporte a la Ciencia de la Implementación, especialmente en el ámbito de la eHealth, ya que, se considera que la evaluación de esta iniciativa proporciona información valiosa para optimizar la implementación de futuras intervenciones en la práctica clínica.

Adicionalmente, este estudio agrega otro potencial aporte al utilizar el CFIR 9 como guía para la investigación, tal como otros estudios que han mostrado que este marco es una herramienta útil para la identificación de factores que influyen en el éxito o fracaso de la implementación de una nueva intervención 17,20,30,34,38-39.

De acuerdo con lo reportado, fueron abordados principalmente constructos correspondientes a los dominios: características de la intervención, contexto interno y proceso de implementación, los cuales coinciden con estudios sobre el uso de intervenciones en eHealth en pacientes con enfermedades crónicas 20,23,34-35. Por otra parte, y en concordancia con otras investigaciones 40, se observa que los cinco dominios se interrelacionan y pueden llegar a sobreponerse, por lo que se sugiere analizarlos inicialmente de manera independiente para luego dar paso a un análisis integrado.

### Limitaciones del estudio

La recolección de información se llevó a cabo después de tiempo transcurrido desde que se implementó el proyecto piloto de telemonitoreo, por lo que la información brindada por los informantes clave se vio afectada por el sesgo del recuerdo. A ello se suman las dificultades para acceder a registros oficiales que documenten el proceso de implementación. Si bien el CFIR brinda un sofisticado y factible aporte para la investigación de la implementación, su utilización en la práctica puede resultar compleja dada su extensa lista de constructos.

A través del uso del CFIR se identificaron las principales barreras del proyecto piloto de telemonitoreo: complejidad, adaptabilidad, recursos disponibles, planificación, ejecución y evaluación de la intervención. Mientras que sus principales facilitadores fueron la ventaja relativa, la identificación de las necesidades del paciente y los conocimientos y las creencias del equipo gestor involucrado. La evaluación y la identificación de barreras y facilitadores en la implementación de las intervenciones de eHealth es crucial para asegurar su efectividad, optimizar el uso de recursos, promover el aprendizaje continuo y respaldar la toma de decisiones informadas, maximizando los beneficios para los pacientes, los profesionales de la salud y los sistemas de atención médica en general ♦
